# Consequences of reaming with flat and convex reamers for bone volume and surface area of the glenoid; a basic science study

**DOI:** 10.1186/s13018-015-0312-7

**Published:** 2015-11-25

**Authors:** Anne Karelse, Steven Leuridan, Alexander Van Tongel, Philippe Debeer, Jos Van Der Sloten, Kathleen Denis, Lieven F. De Wilde

**Affiliations:** Department of Orthopaedic Surgery and Traumatology, Ghent University Hospital, De Pintelaan 185, 9000 Gent, Belgium; Department of Mechanical Engineering, Biomechanics Section, Catholic University of Leuven, Celestijnenlaan 300C, 3001 Heverlee, Belgium; Department of Orthopaedics, University Hospital Pellenberg, Weligerveld 1, B-3212 Pellenberg, Belgium; Catholic University of Leuven, Celestijnenlaan 300C, 3001 Heverlee, Belgium; Catholic University of Leuven, Andreas Vesaliusstraat 13, 3000 Leuven, Belgium

**Keywords:** Glenoid, Reaming, Erosion, Version, Shoulder, Prosthesis

## Abstract

**Background:**

The effect of reaming on bone volume and surface area of the glenoid is not precisely known. We hypothesize that (1) convex reamers create a larger surface area than flat reamers, (2) flat reamers cause less bone loss than convex reamers, and (3) the amount of bone loss increases with the amount of version correction.

**Methods:**

Reaming procedures with different types of reamers are performed on similar-sized uniconcave and biconcave glenoids created from Sawbones foam blocks. The loss of bone volume, the size of the remaining surface area, and the reaming depth are measured and evaluated.

**Results:**

Reaming with convex reamers results in a significantly larger surface area than with flat reamers for both uniconcave and biconcave glenoids (*p* = 0.013 and *p* = 0.001). Convex reamers cause more bone loss than flat reamers, but the difference is only significant for uniconcave glenoids (*p* = 0.007).

**Conclusions:**

In biconcave glenoids, convex reamers remove a similar amount of bone as flat reamers, but offer a larger surface area while maximizing the correction of the retroversion. In pathological uniconcave glenoids, convex reamers are preferred because of the conforming shape.

## Background

**Fig. 1:**
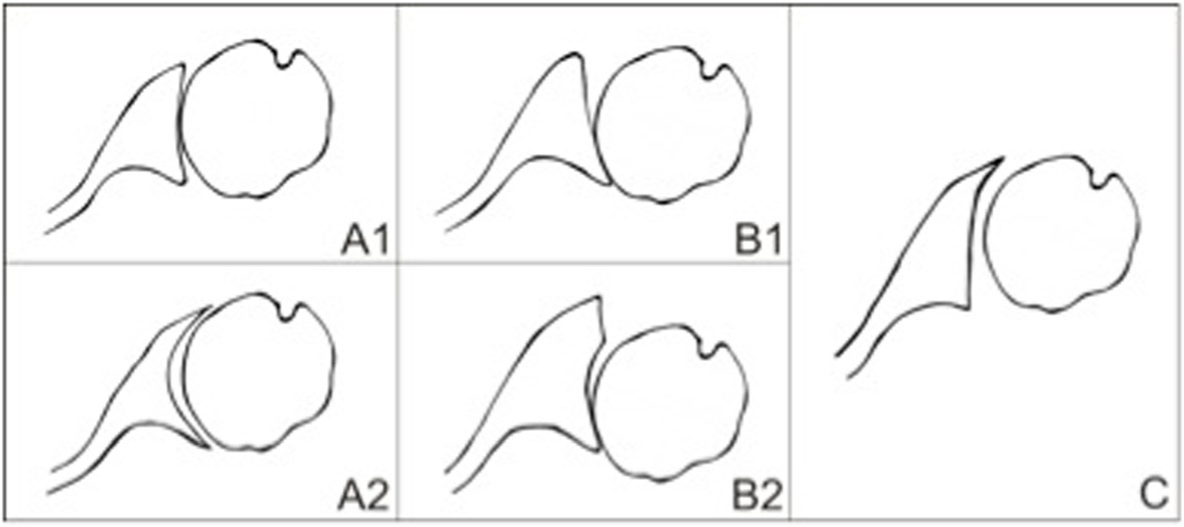
Walch classification: in type A glenoids, the humeral head is centered and the erosion is central. The severity of the erosion is either minor (*A1*) or major (*A2*). In type B, there is asymmetric posterior wear of the glenoid associated with posterior subluxation of the humeral head. In type *B1*, the erosion is minor with joint space narrowing, subchondral sclerosis, and osteophytes. In type *B2*, the erosion is major and the glenoid has become biconcave. Type *C* is defined as a dysplastic glenoid with retroversion of more than 25°; the head remains centered

Glenohumeral osteoarthritis is often associated with glenoid bone deformation and deficiency due to chondral and bone erosion. The erosion is concentric in approximately 60 % and eccentric in over 30 % according to Walch et al. [1] (Fig. 1). In total shoulder arthroplasty, the increased retroversion and erosion of the glenoid are associated with a higher rate of loosening of the glenoid component [2, 3]. Optimal positioning of the glenoid prosthesis seems to be essential to achieve good long-term results. To obtain this, the surgeon should aim to correct the retroversion, while minimizing glenoid bone loss and creating a maximal and congruent contact surface area to support the prosthesis [2, 4, 5]. In glenoids with concentric erosion, type A according to Walch, this brings few difficulties. With limited reaming, a congruent surface with a maximum contact area of the supporting bone offers optimal initial stability to the implant. In contrast, in type B glenoids with eccentric erosion, this cause more problems [6, 7]. It has been suggested to correct the retroversion to as close to the native version as possible (to within 5°); however, the exact amount of correction has not been clearly defined. Eccentric downreaming can correct less severe retroversion, but the amount of reaming is limited by the glenoid bone volume and by the medialization of the joint line.

It is not known how much bone is exactly removed by reaming or how this reaming affects the glenoid supporting area with respect to the pathology of the glenoid. The purpose of this study is to quantify bone loss and contact surface area of uniconcave and biconcave glenoids after reaming with different types of reamers. We hypothesize that (1) convex reamers create a larger surface area than flat reamers, (2) flat reamers cause less bone loss than convex reamers, and (3) the amount of bone loss increases with the amount of version correction.

## Methods

### Bone models

**Fig. 2:**
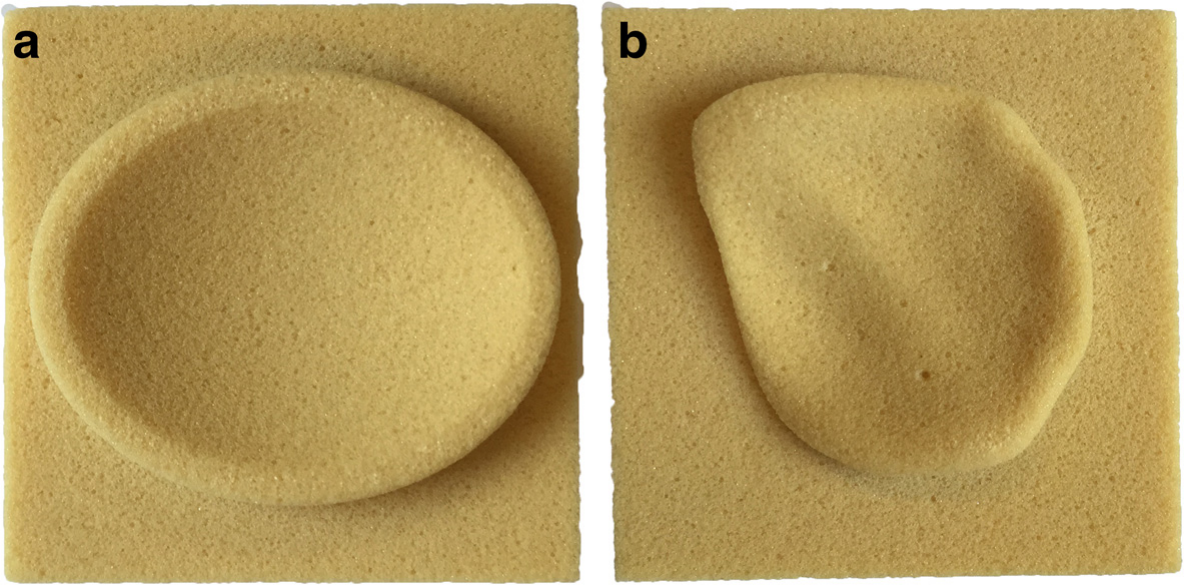
**a** Uniconcave type A glenoid bone block. **b** Biconcave type B2 glenoid bone block

Seventy-two glenoid bone models were created from Sawbones foam blocks: 36 with a uniconcave shape and 36 with a biconcave shape, hereby mimicking type A and type B2 glenoids according to the Walch classification [1]. The Sawbones solid rigid polyurethane foam (Sawbones, Malmo, Sweden) has material properties similar to the subchondral glenoid bone [8]. The dimensions of an original female biconcave glenoid (82-year-old woman with glenohumeral osteoarthritis) were used to create the B2 glenoid models. The dimensions of the B2 glenoid were obtained from a CT scan of the glenoid. The radius of the inferior circle of the native glenoid was 15 mm [9, 10]. The retroversion measured according to Friedman et al. [11] was 12°. From this CT scan, an Standard Tesselation Language (STL) surface was extracted using Mimics (Materialise, Haasrode, Belgium), which was used to prepare the computer-aided design (CAD) drawings and generate computer-aided manufacturing (CAM) commands for the milling process in NX 7.5 (Siemens PLM, Plano, TX, USA). In this way, the STL surface of the patient was replicated onto the Sawbones blocks. The A model glenoids were ovoid in shape [12, 13] and were not CT-based, but were chosen comparable in size to the B models, measuring 30 mm in width, 39 mm in length, and with a depth of 5 mm. The version is neutral, 0°. Again, starting from a STL surface of the CAD drawing, CAM commands were generated for the milling process in NX 7.5 (Siemens PLM, Plano, TX, USA). Both type A and B2 glenoids were milled from the polyurethane blocks using a three-axis milling machine (Haas, Oxnard, CA, USA) (Fig. 2a, b). Automation of the milling process ensured that all fabricated glenoid blocks were exact within manufacturing tolerance (10 μm).

### Methodology

**Fig. 3:**
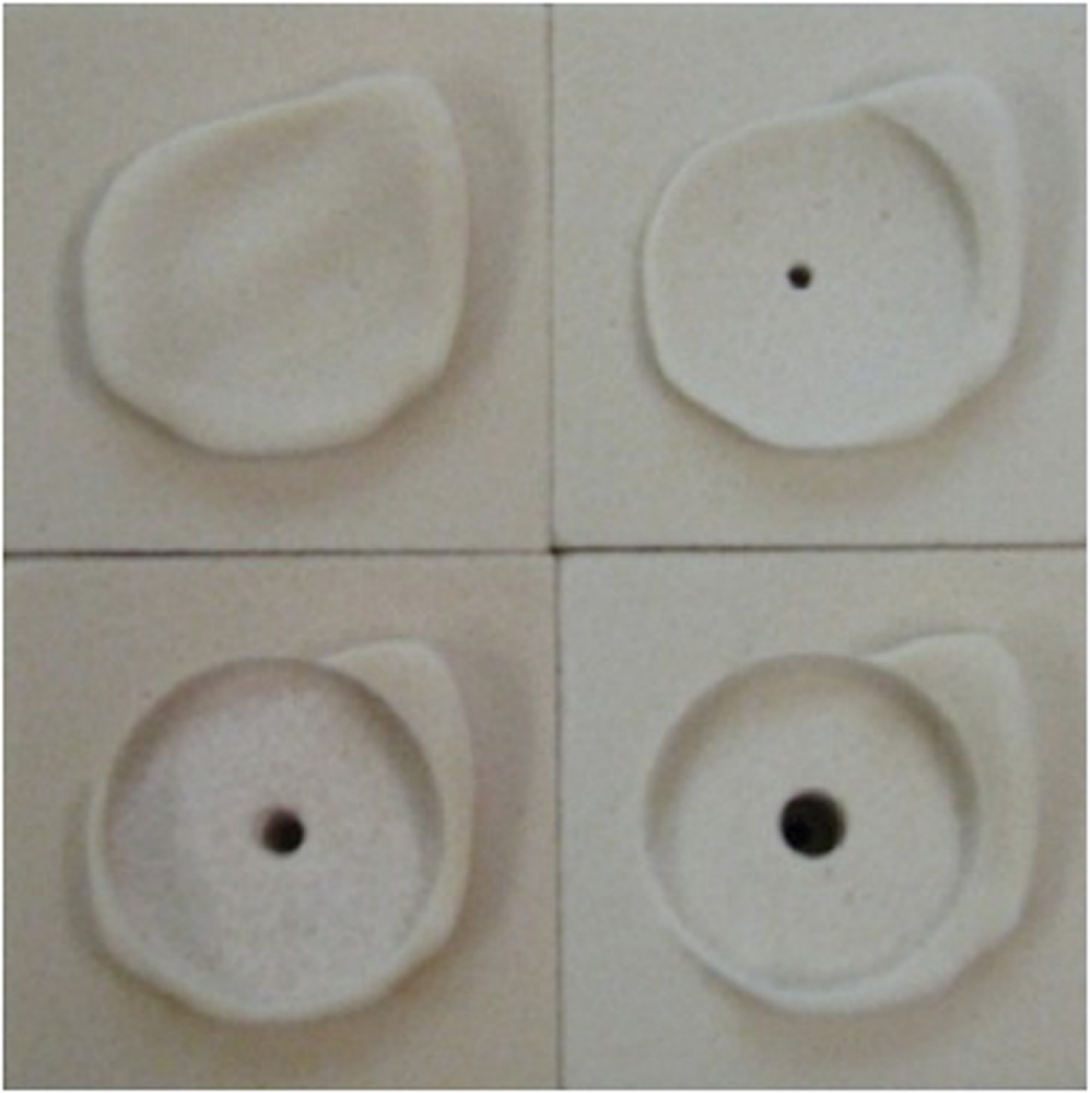
Reamed B2 glenoids

A setup is prepared with the bone blocks positioned vertically at surgical working height. The reaming procedure we used was validated in 2013 [14]. Three surgeons and three authors perform the reaming representing an experienced, intermediate, and inexperienced surgeon with respectively over 50, over 20, and under 20 total shoulder arthroplasties performed per year. Each surgeon individually defines the preferred center of the glenoid for the reaming procedure using a flat semicircular guide (Zimmer, Warsaw, IN). The surgeons individually determine the direction of reaming to obtain the intended correction of the version. For the A glenoids, the aim is to keep the version neutral. The B2 glenoids have a retroversion of 12°, and the aim is to correct this as close to neutral as possible. For the A glenoids, reaming is performed until the reamer is over its entire surface in contact with the glenoid bone, creating a smooth bone bed. For the B2 glenoids, reaming is performed similarly taking into account a correction to a neutral version (Fig. 3). Four different reamers with the same radius are used: a convex reamer guided by a K-wire (Global AP, diameter 33 mm, Depuy, Warsaw, Indiana), a convex reamer guided by a nipple (Global Advantage, diameter 33 mm, nipple 6 mm, Depuy, Warsaw, Indiana), a flat reamer guided by a K-wire (custom made, diameter 30 mm), and a flat reamer guided by a nipple (diameter 30 mm, nipple 6 mm, Zimmer, Warsaw, Indiana). All reamers are used with the companies’ instruments with a set arm length (18 cm). Each surgeon reams three A and three B2 glenoids with the four different reamers. This results in 24 reaming procedures per surgeon, 72 all together.

### Parameters

**Fig. 4:**
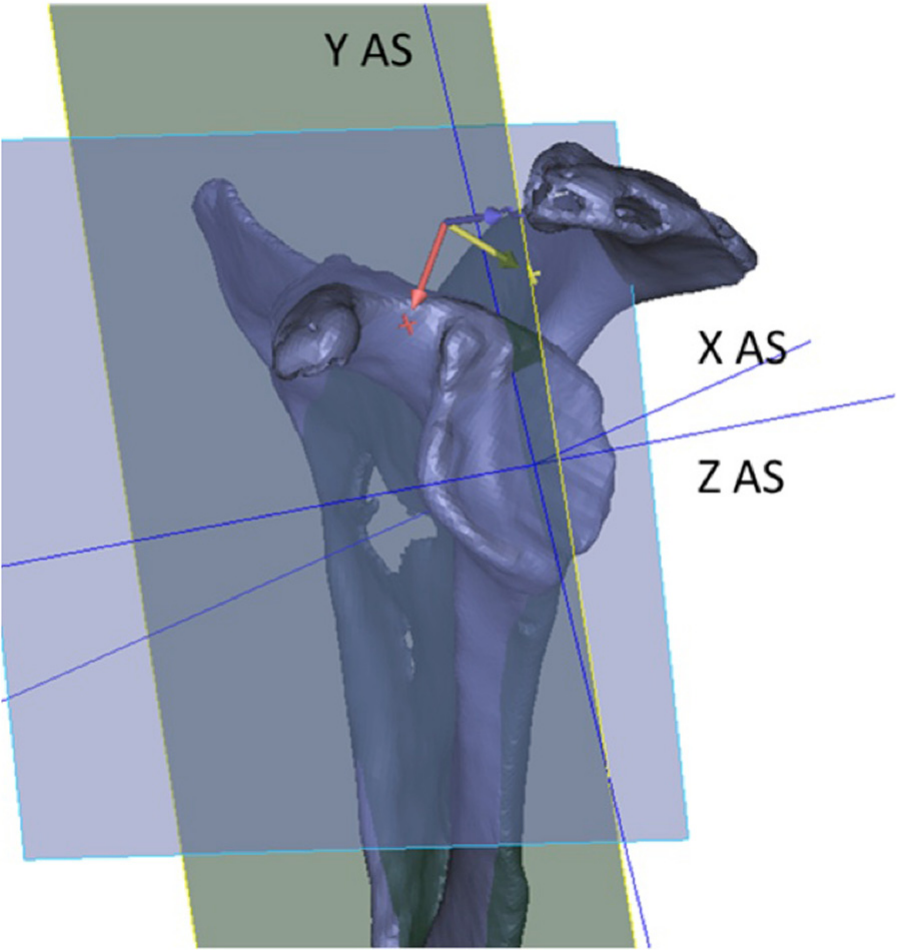
Anatomical coordinate system with *X*- and *Y*-axes in the plane of the glenoid and the *Z*-axis perpendicular to this plane

All bone blocks are scanned using 3D laser coordinate measuring machine (CMM) (MC16, Coord3, Turin, Italy) before and after reaming. The 3D CMM uses a laser to scan the surface of the blocks resulting in a dense point cloud of points lying on that surface. These resulting point clouds are processed using GOM Inspect (Braunschweig, Germany), and STLs are built in 3-matic (Materialise, Haasrode, Belgium) based on these point clouds. These STLs are used in the further analyses. All bone models are aligned in the software to the same identical coordinate system (“world coordinate system”), ensuring comparability between the parameters of different blocks. The parameters extracted for all reamed bone blocks are the loss of bone volume, the size of the remaining surface area, and the reaming depth. The bone volume removed is calculated based on the STLs of the respective block taken before and after reaming, similar to the procedure described by Youngpravat et al. [15]. The three direction angles are calculated with respect to the local anatomical *X*-, *Y*-, and *Z*-axes (“anatomical coordinate system”) of the A and B2 glenoids defined according to Verstraeten et al. [16] (Fig. 4). Defining the angles with respect to this, local anatomical coordinate system allows for a uniform and clinically relevant interpretation of the angles for A and B2 blocks. Repeatability of the parameter extraction procedure is verified for all parameters with ten repetitions per parameter and results in a mean standard deviation of 0.23 % on the parameter values.

### Statistics

Statistical analyses are performed using IBM SPSS Statistics, version 21 (SPSS Inc., Chicago, IL, USA). Hypothesis testing between two groups is performed using a *t* test if both groups to be compared were normally distributed according to a Shapiro-Wilk test or using a Mann-Whittney *U* test if one of the groups failed to pass the normality test. When more than two levels per factor are compared, an ANOVA analysis is carried out if the normality assumption is satisfied or a Kruskal-Wallis test if this assumption is not fulfilled. Regression analyses are carried out to assess the relationship between continuous parameters (e.g., direction angles) and relevant outcomes (e.g., bone loss). Significance is assessed at the 5 % level.

## Results

### A glenoids

**Fig. 5:**
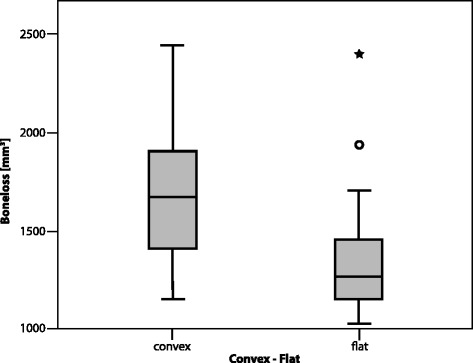
Bone loss in A glenoids for convex and flat reamers

**Fig. 6:**
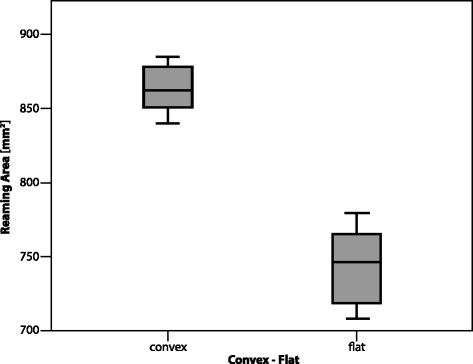
Surface area in A glenoids for convex and flat reamers

Convex reamers cause significantly more bone loss than flat reamers (*p* = 0.007) (Table 1, Fig. 5). Reaming with convex reamers results in a significantly (*p* = 0.013) larger surface area than flat reamers (Table 2, Fig. 6) and a significantly greater average depth of reaming (*p* < 0.001). We find no significant difference in bone loss (*p* = 0.174), surface area (*p* = 0.521), and depth (*p* = 0.278) between reaming with a K-wire or a nipple-guided reamer, for both flat and convex reamers. The regression between bone loss and the three direction angles is not significant (*p* = 0.4). Hence, none of the three direction angles show a significant relation to the bone loss: *X* direction angle (*p* = 0.566), *Y* direction angle (*p* = 0.108), and *Z* direction angle (*p* = 0.568). The regression between the surface area and the three direction angles is not significant (*p* = 0.058): *X* direction angle (*p* = 0.083), *Y* direction (*p* = 0.070), and *Z* direction (*p* = 0.219). The regression of bone loss to the depth of reaming shows a significant relation (*p* < 0.001, *R*^2^ = 0.469). Every millimeter of additional reaming depth accounts for an extra 215 mm^3^ of bone loss for the given A glenoid samples.

**Table 1:** Bone loss in A and B2 glenoids for convex and flat reamers

Reamer	Glenoid	No.	Bone loss (mm3)	
			Mean	SD
Convex	A	18	1686	352
Flat	A	18	1381	343
Convex	B2	18	1779	447
Flat	B2	18	1807	473

**Table 2:** Surface area in A and B2 glenoids for convex and flat reamers

Reamer	Glenoid	No.	Surface area (mm2)	
			Mean	SD
Convex	A	18	864	14
Flat	A	18	744	24
Convex	B2	18	794	38
Flat	B2	18	730	42

### B2 glenoids

There is no significant difference in bone loss between flat and convex reamers (*p* = 0.855). Reaming with convex reamers results in a significantly larger surface than with flat reamers (*p* = 0.001). The average depth of reaming is significantly greater with convex reamers than with flat reamers (*p* < 0.001). There is no significant difference in bone loss (*p* = 0.174) and depth (*p* = 0.449) between reaming with a K-wire or a nipple-guided reamer, both for flat and convex reamers. A significant difference however exists in the reaming area (*p* < 0.001) between reaming with a K-wire or a nipple-guided reamer for flat reamers. No significant difference in reaming area is recorded for K-wire versus nipple-guided wires for convex reamers (*p* = 0.529). The regression between bone loss and the *X* direction angle is significant (*p* = 0.002, *R*^2^ = 0.249). The regression coefficient shows that every angle degree of correction along the *X*-axis results in an additional bone loss of 56 mm^3^ for the B2 bone samples used. The *Y* direction angle (*p* = 0.943) and the *Z* direction angle (*p* = 0.288) show no significant relation to the bone loss. There is a significant difference in reaming angle between surgeons 1 and 2 for the *X* direction angle (*p* = 0.14) and between surgeons 1 and surgeon 3 (*p* < 0.001). No significant difference can be found between surgeons 2 and 3 (*p* = 0.296). Surgeon 3 corrects to an average *X* direction angle of 83.9° (±2.257), surgeon 2 corrects to an average *X* direction angle of 85.67° (±3.33), and surgeon 1 corrects to an average *X* direction angle of 89.58° (±3.83°). The regression between the surface area and the three direction angles is not significant (*p* = 0.817): *X* direction angle (*p* = 0.459), *Y* direction (*p* = 0.792), and *Z* direction (*p* = 0.856). The regression of bone loss to the depth of reaming shows a significant relation (*p* = 0.001, *R*^2^ = 0.290). Every millimeter of additional reaming depth accounts for an extra 235 mm^3^ of bone loss for the given B2 glenoid samples.

## Discussion

Glenoid component failure remains the most important indication for revision surgery of total shoulder arthroplasty [17–19]. Biomechanical studies have shown that placement of a glenoid component in more than 10° of retroversion causes eccentric loading of the prosthesis, and this can lead to instability, rocking horse phenomenon, and early loosening [7, 20–24]. Correction of the version helps to restore the glenohumeral relationship and rebalances the force couple of the rotator cuff. Downreaming of the anterior glenoid is an accepted method to correct the retroversion, but limited by the volume of the glenoid vault. Excessive reaming can result in loss of glenoid bone stock and medialization of the joint line jeopardizing solid fixation and with the risk of peg perforation [25–27]. If the retroversion is less than 15° to 20°, downreaming of the anterior glenoid is advised. However, there are no explicit guidelines regarding the amount of version that can be safely corrected by eccentric reaming without compromising the glenoid bone stock [28]. We do know that there are limits and clinical experience proved reverse shoulder arthroplasty to be a viable surgical option to solve both the problem of severe glenoid erosion in patients with a biconcave glenoid without rotator cuff insufficiency [29].

The amount of bone resected by the different types of reamers (nipple or K-wire guided, flat and convex) is unknown. To our knowledge, this is the first study investigating the effect of reaming with different reamers on bone volume and surface area in two different-shaped glenoids. This study shows that convex reamers cause more bone loss than flat reamers in uniconcave type A glenoids. This is partly due to the deeper reaming range as a result of the convexity. Corrective reaming of biconcave type B2 glenoids with convex reamers tends to cause slightly more bone loss than with flat reamers, but the difference is not significant. In A glenoids, the reaming angle is as close to neutral as possible, so this does not interfere with bone loss. The depth of reaming does have a significant effect on bone loss, and every millimeter of additional reaming depth accounts for an extra 215 mm^3^ of bone loss. In B2 glenoids, the angle of correction along the *X*-axis (representing the version angle correction) is an important factor in determining the bone loss; every additional degree of correction along the *X*-axis results in an extra 56 mm^3^ of bone loss. Similarly, the depth of reaming has an important effect on bone loss; every millimeter of additional reaming depth accounts for an extra 235 mm^3^ of bone loss. Obviously, it is the degree of retroversion and biconcavity, and the intended correction, which dictates the loss of bone volume after reaming in biconcave glenoids. If a surgeon decides to correct more by reaming, this has a direct effect on the amount of bone loss. There is a significant difference between surgeons in the correction of version in the B2 glenoids in this study. This is probably due to the surgeons’ intention and experience to correct as close as possible to the native version [15]. In recent publications, Iannotti et al. [30] and Karelse et al. [14] came to a similar conclusion that in biconcave glenoids, correction of version by reaming is not reproducible. Until now, corrective reaming is performed by “carpenters eye,” helped by the experience and the natural 3D orientation of the surgeon. Several studies show that the accuracy of the position of the glenoid prosthesis in the transverse plane can be improved by intraoperative navigation and patient-specific instrumentation; this is particularly so in severely retroverted glenoids [31–35]. Convex reamers create a larger surface area than flat reamers in both A and B2 glenoids, and this is not affected by the correction angle. This finding differs from the results from Youngpravat et al. [15], where smaller version corrections increase the surface area. A larger surface area obviously increases the contact area for a glenoid component. In biconcave glenoids, the convex reamers are at slight disadvantage to flat reamers concerning bone loss, but they win back in a larger surface area of the glenoid after reaming. For uniconcave type A glenoids, which are considered non-pathological glenoids, reaming with convex reamers causes more bone loss than with flat reamers. The difference in surface area between the reamers is small given the fact that reaming depth must be minimal in these non-eroded glenoids. If however glenoids are centrally eroded to type A1 and A2 glenoids according to Walch, and excessive medialization of the joint line should be avoided, minimal reaming with a more conforming reamer is the objective. A convex reamer with a radius of curvature mimicking the radius of the native articular surface can maximally preserve the surface area and existing bone stock in centrally eroded glenoids [36], whereas flat reamers would reduce both surface area and bone stock. Another explanation for the reduced bone loss after flat reaming can be that the radius of flat reamers is chosen accordingly to the largest radius of the glenoid, thereby reaming mainly the circumferential bone and not reaching the centrally eroded part.

The surface area of B2 glenoids is larger after reaming over K-wires than nipple guided using flat reamers. The difference may partly be explained by the difference in diameter of the K-wire and the nipple, 2 and 6 mm, respectively.

We are aware of the limitations of this study. We performed reaming procedures on foam blocks in a surgical setup but without the intraoperative conditions that can be of great influence to a procedure. We created only two types of morphology while we are aware of the large variation of the concavity of the glenoid. Nevertheless, we believe that this study offers valuable information that can be of help in future decisions on reaming strategy and possibly influence the choice and development of flat or curved backed glenoid prostheses for certain pathological glenoids [36–39].

## Conclusion

This study shows that the characteristics of the reamer and the experience of the surgeon influence the amount of bone removal and the remaining surface area of the glenoid. These findings account for the two morphologic types studied: A and B2 glenoids. Convex reamers are due to their conforming shape that is better indicated in pathological A glenoids, but the convexity of the reamer should be optimally adapted to the pathological curvature [38]. In B glenoids, convex reamers are preferred because they remove a similar amount of bone as flat reamers but offer a larger surface area while maximizing the correction of the retroversion.
